# Cecal Volvulus: A Report of a Challenging Case

**DOI:** 10.7759/cureus.45753

**Published:** 2023-09-22

**Authors:** Ahmed Al Khalifa, Alaa Sedik

**Affiliations:** 1 College of Medicine, Sulaiman Alrajhi University, Al Bukayriyah, SAU; 2 General Surgery, King Khalid General Hospital, Hail, SAU

**Keywords:** lactic acidosis, right hemicolectomy, ct -scan, ileostomy, cecal volvulus

## Abstract

Cecal volvulus represents a rare form of acute intestinal obstruction caused by an axial twist of the terminal ileum, ascending colon, and cecum surrounding the mesenteric pedicle. It is responsible for 1%-1.5% of all intestinal obstruction cases in adults. Radiological imaging assists in the diagnosis of cecal volvulus, particularly a CT scan with contrast as the gold standard for both diagnosis and risk assessment.

In this case report, we present a challenging case of cecal volvulus seen in a 75‑year‑old male patient with multiple comorbidities who presented with abdominal guarding/tenderness and high WBC and lactate, which evolved into septic shock. The purpose of this study is to underline the significance of early diagnosis and effective treatment of this uncommon condition in abdominal surgeries.

## Introduction

Cecal volvulus is the twisting of movable cecum and ascending colon, which accounts for 1 to 3% of all significant bowel obstruction cases. Cecal volvulus, if left untreated, can lead to bowel necrosis, perforation, or ischemia. Although volvulus can develop in other parts of the gastrointestinal tract, such as the small bowel, gallbladder, and stomach, it is more frequent in the colon. The cecum and sigmoid colon are the two most prevalent locations of the volvulus [1].

The symptoms of cecal volvulus may include abdominal distension, constipation, severe abdominal pain, and vomiting. Cecal volvulus can be difficult to diagnose since it shares a similar presentation with other etiologies of intestinal obstruction. These symptoms are sometimes misdiagnosed as irritable bowel syndrome (IBS) or inflammatory bowel disease (IBD). Imaging investigations, in addition to a physical exam, can assist in identifying cecal volvulus. A CT scan or an X-ray can be used for the diagnosis, and surgery is the mainstay approach to managing cecal volvulus. If treatment is delayed, cecal volvulus has a mortality rate of up to 30% because of the increased death rate and associated complications. Even in stable patients, early surgical intervention should be provided if clinical suspicion exists [2].

This case report describes a 75-year-old male patient having multiple morbidities diagnosed as a case of cecal volvulus and managed by a right hemicolectomy with both ends left stapled for a second look during which he was still unstable and a terminal Ileostomy was fashioned.

## Case presentation

A 75-year-old male patient was brought to the ER for a decreased level of consciousness and a documented fever for the last four days. He had a history of diabetes mellitus, hypertension, chronic kidney disease on regular hemodialysis, and a cerebrovascular accident a few months back but not on regular medications. He has had a convulsion on admission on a background of multiple strokes.

On general examination, the patient had a Glasgow Coma Scale (GCS) of 10/15, his vital signs were abnormal, after being supported with intravenous fluids and inotropes. Vital signs were a pulse rate of 95 beats per minute, blood pressure of 108/50 mmHg, afebrile, and oxygen saturation of 97% on atmospheric oxygen, His right dorsalis pedis was weak but palpable. The patient was disoriented and had residual weakness on his right side; he had neck stiffness, tremors, and pain aggravated on elevating his legs. He had an ischemic ulcer on his right foot with no symptoms of infection. On abdominal examination, the abdomen was slightly distended and there was abdominal guarding and tenderness.

Laboratory investigations showed hemoglobin of 8.50 g/dl, white blood cell count of 11.8 /mm^3^, sodium of 147 mmol/l, potassium of 4.5 mmol/l, and an estimated sedimentation rate (ESR) of 145 mm/hr. The patient had a positive blood culture of staphylococcus aureus. Lactate, urea, and creatinine were abnormally high.

**Figure 1:**
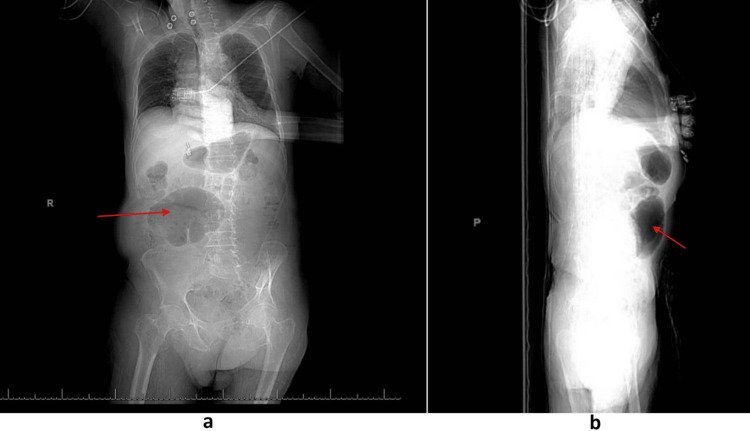
X-ray of the abdomen showing a hugely dilated loop of bowel with haustra filled with gas slightly to the right of the midline a) anterior-posterior view; b) lateral view

CT scan of the brain showed old infarcts. Abdominal X-ray showed multiple dramatic dilatations of the bowel loops filled with gas (Figure 1).

**Figure 2:**
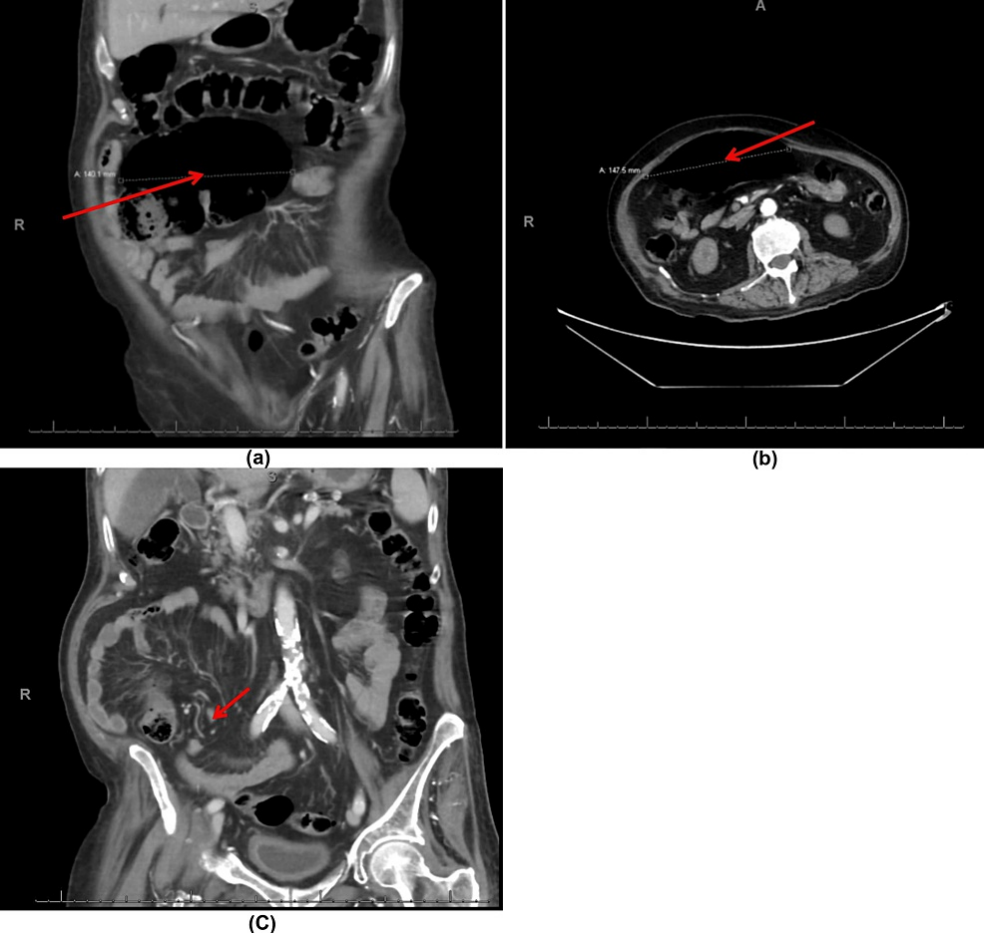
CT abdomen with IV contrast showing a twist in the bowel, mesentery, and associated vessels ''whirlpool sign'', and a grossly dilated cecum of 14 cm diameter (a) Coronal view showing the hugely dilated cecum; (b) Transverse view; (c) Coronal view showing the ''whirlpool sign'' with torsion of the cecal vessels

CT abdomen with IV contrast showed a picture of malalignment of the cecum that is seen markedly distended about 14 cm directing upwards and medially with swirling of the feeding vessels (Figure 2).

A provisional diagnosis of a cecal volvulus was made and after resuscitation with intravenous fluid, inotropes, Foley catheter insertion, and nasogastric tube placement the patient was shifted to the operating room.

**Figure 3:**
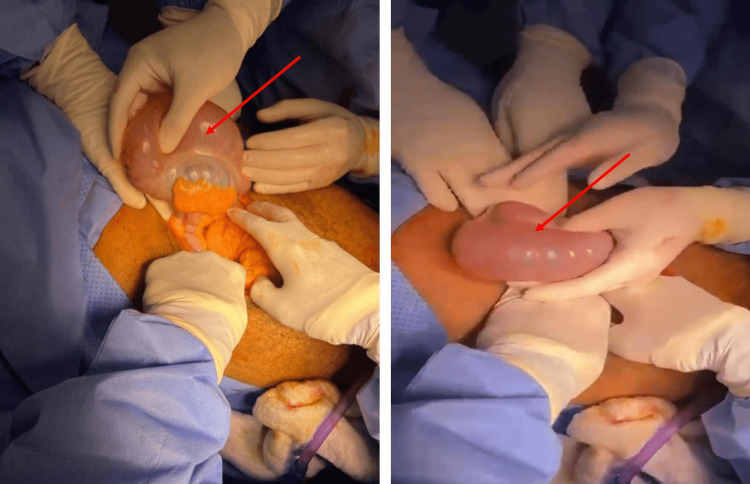
Intraoperative discoveries showed a type 2 organoaxial cecal volvulus

An emergent exploratory laparotomy through a mid-line incision was done on 04-06-2023. Intraoperatively, a 360 degrees anticlockwise volvulated mobile cecum was discovered (Figure 3), the viability was questionable, and many serosal tears due to the distension were identified.

Due to the instability of the patient's condition, and the compromised viability of the volvulus, a right hemicolectomy was done and both ends left stapled for a possible second look. One-unit packed RBCs was transfused during the operation and a temporary abdominal closure of skin-only with continuous vertical mattress with proline No. 1 suture was done. The patient came back from the operating room to the ICU intubated.

Histology showed a right hemicolectomy specimen with twisting at the cecal area with submucosa and serosa showing dilated vessels occluded by thrombus consistent with cecal volvulus.

A second-look laparotomy with terminal ileostomy was performed on 06-06-2023, a right pelvic drain was placed and skin-only closure with interrupted vertical mattress using proline No. 1 was performed. After this, the patient was shifted back to ICU due to his unstable condition. after being placed on NPO, the patient was gradually started on NGT feeding on 08-06-2023. The patient had an excessive secretion in the chest thus he was recommended for suctioning and chest physiotherapy. He was then shifted to TPN and partial NGT feeding. Surgical drainage showed bloody fluid. Abdomen and pelvic ultrasound showed a localized fluid collection at the lateral aspect of the right iliolumbar region, measuring 7x2.5cm. NGT wash showed a small amount of blood clots. The patient was on mechanical ventilation through an orotracheal tube in CMV mode. Difficulty weaning off mechanical ventilation was encountered because of the patient’s central apnea due to his multiple comorbidities including previous strokes, old age, and renal failure.

On postoperative examination, the abdominal assessment showed right flank tenderness to palpation, right pelvic drain, and multiple ecchymoses were noted, with a functioning stoma. Medically, the patient was in an unfavorable condition, had many old strokes, coagulopathy, uncontrolled hyperglycemia, and was hemodynamically impaired. On follow-up, the norepinephrine requirement decreased. The patient was advised to discontinue sedation, analgesia, and extubation. Following his release from the vent, he experienced airway protection issues in the form of thick secretion and an oral roof pressure ulcer due to a viral infection. The patient was on meropenem and oxacillin for a positive blood culture of methicillin-susceptible Staphylococcus aureus. He had low platelets and had a transfusion of 5 units of platelets. He was considered for catheter removal. He was then admitted to IMCU. Due to his multiple comorbidities, and deterioration in vitals, the patient was referred back to ICU care and passed away in the ICU.

## Discussion

Volvulus occurs when segments of the colon get entangled along a mesenteric axis, resulting in impaired blood flow and a total or partial obstruction of the intestinal lumen. Globally, colonic volvulus is the third most common cause of colon obstruction, after cancer and diverticulitis [3].

Although it can occur in either the large or the small bowel, the most common areas in adults are the sigmoid and ileocecal areas. The sigmoid colon (80%), cecum (15%), transverse colon (3%), and splenic flexure (2%) are the most prevalent sites of large-bowel volvulus [4]. The incidence of cecal volvulus is reported to range from 2.8 to 7.1 per million people per year, and the process is responsible for 1%-1.5% of all adult intestinal obstructions and 25%-40% of all volvulus involving the colon [5].

Risk factors for cecal volvulus include previous abdominal surgeries, pregnancy (especially in the third trimester), following a high-fiber diet, having pelvic masses, taking psychotropic medications, and chronic constipation [6]. Cecal volvulus can be classified into three categories. Approximately 80% of all cecal volvuli are type 1 and type 2, which entail axial torsion. The remaining 20% of cecal volvuli are made up of cecal bascules [7].

The clinical presentation varies widely, ranging from sudden intermittent episodes of abdominal pain to an acute abdominal emergency. In addition, patients may also experience nausea, vomiting, and constipation [8]. The duration of symptoms can fluctuate, ranging from a few hours to several days [9].

Laboratory findings can be helpful, but they are not diagnostic, a complete blood count (CBC) with differential, a thorough metabolic panel, and a lactic acid test are all part of the workup for a colonic volvulus [10]. A high degree of leukocytosis, a left shift, or metabolic acidosis might suggest systemic sepsis, intestinal perforation, or peritoneal infection, However, none of these altered parameters are specific for cecal volvulus [11]. In this case report, the patient experienced leucocytosis and lactic acidosis.

Radiographic imaging can assist in the differentiation of sigmoid and cecal volvulus from other abdominal disorders such as abdominal hernias, appendicitis, acute mesenteric ischemia, Iliosigmoid knot, sigmoid diverticular disease, or pseudo-obstruction. An abdominal x-ray demonstrates a dilated gas-filled cecum with an air-fluid level that can be displaced anywhere in the abdomen depending on the volvulus type. This intestinal loop may indicate a twisted cecum, with the caput cecum oriented medially [12].

Definitive treatment should be sought as soon as the patient has been adequately resuscitated, Initial management entails conservative measures such as analgesia, antiemetics, intravenous fluids, having the patient stop any oral intake, insertion of a nasogastric tube to facilitate proximal bowel decompression, and insertion of a urinary catheter to monitor fluid input/output [13].

Surgical intervention is the primary management of cecal volvuli, surgical options are determined by the hemodynamic stability of the patient and the presence or absence of bowel compromise [14]. Other nonoperative reduction options (e.g., by colonoscopy or barium enema) are rarely successful (<5 percent) and could cause perforation; it therefore should not be attempted [15]. In our case, given that the involved segment's viability had been compromised and the patient was hemodynamically unstable, right hemicolectomy followed by ileostomy rather than a primary ileocolic anastomosis was performed. considering the patient's critical instability a temporary abdominal closure was applied in both surgeries.

Even after treatment for cecal volvulus, patients have significant morbidity due to prolonged ileus, wound infection, respiratory failure, and intestinal obstruction. Patients frequently require a long hospital stay. The majority of patients are old and feeble. If the ileus is severe, they may require IV fluids for many days. Physical therapy and DVT prevention are frequently advised [16]. In this report, the patient was advised to undergo DVT prophylaxis as well as chest and limb physiotherapy.

## Conclusions

This case report presents a complex case of a patient with cecal volvulus with multiple comorbidities. Cecal volvulus is an uncommon cause of intestinal obstruction. Because the symptoms and indications are nonspecific, early diagnosis is dependent on a high index of suspicion. The increased use of CT scans in individuals with intestinal obstruction has improved early diagnosis. To avoid major consequences, such as intestinal necrosis, cecal perforation, and generalized peritonitis, early diagnosis, prompt resuscitation, and surgical intervention are critical. Surgical methods are determined by a variety of parameters, including intraoperative findings and the patient's stability.
